# Metabolic syndrome is a risk factor for breast cancer patients receiving neoadjuvant chemotherapy: A case-control study

**DOI:** 10.3389/fonc.2022.1080054

**Published:** 2023-01-04

**Authors:** Zhaoyue Zhou, Yue Zhang, Yue Li, Cong Jiang, Yang Wu, Lingmin Shang, Yuanxi Huang, Shaoqiang Cheng

**Affiliations:** ^1^ Department of Breast Surgery, Harbin Medical University Cancer Hospital, Harbin, China; ^2^ Department of Medical Oncology, Harbin Medical University Cancer Hospital, Harbin, China

**Keywords:** breast cancer, metabolic syndrome, neoadjuvant chemotherapy, pathologic complete response, prognosis

## Abstract

**Purpose:**

To investigate the impact of metabolic syndrome (MetS) on pathologic complete response (pCR) and clinical outcomes in breast cancer (BC) patients who received neoadjuvant chemotherapy (NAC).

**Methods:**

We analyzed 221 female BC patients at Harbin Medical University Cancer Hospital who received NAC and divided them into MetS and non-MetS groups according to National Cholesterol Education Program Adult Treatment Panel III (NCEP-ATP III) criteria to investigate the association between MetS and clinicopathological characteristics, pathologic response, and long-term survival and to observe the changes in metabolic parameters after NAC.

**Results:**

A total of 53 (24.0%) BC patients achieved pCR after NAC in our study. MetS status was an independent predictor of pCR, and pCR was more difficult to obtain in the MetS group than the non-MetS group (P=0.028). All metabolic parameters deteriorated significantly after NAC, especially the blood lipid index (P<0.010). The median follow-up time was 6 years. After adjusting for other prognostic factors, MetS was found to be strongly associated with an increased risk of recurrence (P=0.007) and mortality (P=0.004) in BC patients receiving NAC. Compared to individuals without any MetS component, the risk of death and disease progression increased sharply as the number of MetS components increased.

**Conclusions:**

In BC patients who received NAC, MetS was associated with poor outcomes, including a lower pCR rate and increased risks of recurrence and mortality.

## Introduction

In 2020, 19.3 million new cancer cases were diagnosed worldwide, including 2.3 million cases (11.7%) of breast cancer (BC), which has now surpassed lung cancer as the most commonly diagnosed cancer (1). Based on improved and intensified treatments developed over the past few decades, including neoadjuvant chemotherapy (NAC), the BC survival rate has improved significantly (2, 3). NAC has been established as a standard treatment approach in BC patients with locally advanced disease. Currently, the role of NAC has expanded to conversion of inoperable tumors to operable tumors or facilitating breast-conserving therapy (BCT) instead of mastectomy (4, 5), which is also known as tumor downstaging. Moreover, the assessment of tumor response to NAC is a useful tool that provides information on the impact of systemic therapies on BC biology (6). Pathologic complete response (pCR) after NAC serves as a significant surrogate marker that predicts better long-term prognosis (7).

As a significant public health problem worldwide, metabolic syndrome (MetS) is a multifactorial metabolic disease with main components, including obesity, hyperglycemia, dyslipidemia, and hypertension, which was initially linked to cardiovascular diseases (CVDs) (8). Several studies have found that CVD surpasses BC and has become the leading cause of death for BC survivors (9, 10). Accumulating evidence reveals a strong association between MetS and BC (11). MetS and its components are associated with increased risks of BC (12), and in-depth research on the association between MetS and the pathogenesis and prognosis of BC is increasing. Extensive literature has reported that metabolic dysregulation may affect the risk for occurrence, recurrence, and mortality of BC and the onset of additional chronic disease (13, 14). Investigation into the relationship between systemic therapies and MetS in BC survivors also represents an area of research that needs to be urgently addressed. Multiple studies have indicated that metabolic disorders, including overweight, dyslipidemia, and hypertension, are associated with worse pCR to NAC (15–17). However, clinical research on how MetS influences BC patients who receive NAC is currently lacking. This article retrospectively analyzed the clinical data of BC patients who underwent NAC before surgery and observed metabolic changes after adjuvant treatment to investigate the relationship between MetS and the pCR and long-term prognosis of BC patients after NAC and to provide a reference for the treatment of BC.

## Materials and methods

### Patient selection

Our study retrospectively analyzed 221 female BC patients who received NAC and underwent surgery at Harbin Medical University Cancer Hospital between September 2012 and December 2017. Before each treatment, patients signed the “Informed Consent Form for Secondary Use of Medical History Data/Biological Specimens” in our hospital. All procedures involving participants in this study were performed in accordance with Research Committee standards and complied with the 1964 Declaration of Helsinki and other amendments to ethical standards. The following patient inclusion criteria were employed (1): histopathologically confirmed BC by core needle biopsy and (2) preoperative NAC and no radiotherapy or endocrine therapy before chemotherapy. The following patient exclusion criteria were used: (1) patients with distant metastasis; (2) patients with other previous tumors; and (3) patients suffering from other diseases that affect body mass index (BMI), blood pressure, sugar and lipid metabolism or serious physical disease.

### Data collection and biochemical variable determination

Clinical data were collected twice before and after NAC, and all data were collected from electronic medical records by two independent investigators. General clinical data included age, menopausal state, number of births, height, weight, blood pressure, fasting blood glucose (FPG), triglycerides (TG), total cholesterol (TC), high-density lipoprotein cholesterol (HDL-C), and low-density lipoprotein cholesterol (LDL-C). BMI was calculated as body weight (kg) divided by the squared height (m2). Venous blood was taken after 12 hours of fasting, and the blood samples were sent to the Biochemical Laboratory of Medical University Cancer Hospital to detect FPG, TG, TC, HDL-C, and LDL-C.

### Definition of MetS

The diagnosis of MetS was based on the National Cholesterol Education Program Adult Treatment Panel III (NCEP-ATP III) criteria (18). Specifically, MetS was diagnosed if three of the following five criteria were present: obesity (waist circumference>88 cm); FPG≥110 mg/dl (6.1 mmol/L); TG≥150 mg/dl (1.7 mmol/L); HDL-C<50 mg/dl (1.3 mmol/L); and blood pressure≥130/85 mmHg. However, waist circumference was not available in this retrospective review given that this factor was not recorded at screening, so a BMI≥25 kg/m^2^ replaced a waist circumference of 88 cm or more in women. This substitution was validated in previous studies (8, 19, 20) and is consistent with the diagnostic criteria for MetS established by the Diabetes Branch of the Chinese Medical Association in 2004 (21). Patients who met the diagnostic criteria for MetS were included in the MetS group; otherwise, patients were included in the non-MetS group.

### Treatment plan

All patients received NAC before surgery and chose chemotherapy regimens according to modern treatment guidelines and patients’ preferences. The following treatment regimens were noted: 78 cases of AC-T; TA scheme in 48 cases; TAC scheme in 76 cases; TCbH scheme in 7 cases; AC-TH scheme in 3 cases; TH scheme in 7 cases and TCb scheme in 2 cases (A: anthracycline; C: cyclophosphamide; T: taxane, including albumin paclitaxel or docetaxel; Cb: carboplatin; H: trastuzumab). The chemotherapy dose was decided by treatment guidelines and body surface area. One cycle of the chosen regimen was repeated every 3 weeks. All patients received at least three cycles of NAC. Surgery was performed after a rest period of 2-4 weeks after the completion of NAC, depending on the patient’s condition. After surgery, all the enrolled patients received necessary follow-up treatment at Harbin Medical University Tumor Hospital. A total of 71.1% (64 cases) of estrogen receptor (ER)+/progesterone receptor (PR)+ patients and 61.3% (19 cases) of ER+/PR- patients received adjuvant endocrine therapy, and a total of 122 (55.2%) patients received radiation therapy.

### Pathological features, molecular subtypes and pCR

The TNM staging system is based on the eighth edition of the American Joint Committee on Cancer (AJCC). ER, PR, human epidermal growth factor receptor 2 (HER2) and Ki67 status were assessed by immunohistochemical (IHC) staining or *in situ* hybridization (ISH). Luminal A, luminal B, HER2-enriched (HER2-E), and triple-negative molecular subtypes were included in this study. In our study, pCR was defined as no residual invasive disease (with or without ductal carcinoma in situ) in the breast and lymph nodes (ypT0/isN0).

### Follow-up

Patients were regularly followed up at Harbin Medical University Cancer Hospital. Examinations were performed every 6 months during the first 5 years of follow-up and every 12 months thereafter. All patients were followed up until death or the study deadline (May 1, 2022) based on clinical records review and telephone. We defined disease-free survival (DFS) as the time from diagnosis until local, contralateral, and distant disease recurrence as well as secondary primary tumors or death from any cause. Overall survival (OS) was defined as the time from diagnosis to death from any cause or the end of follow-up.

### Statistical analysis

All analyses were conducted with SPSS 26.0 statistical software. Descriptive statistics were reported as frequencies and percentages for categorical variables and as the mean ± standard deviation (SD) or median (interquartile range) for continuous variables. Comparison of patient characteristics between the different groups was performed using the independent T-test or nonparametric test for continuous variables and the chi-squared test or Fisher’s exact test for categorical variables as appropriate. Univariate and multivariate analyses and subgroup analyses of the relationship between clinicopathological features and pCR were performed using logistic regression models and log-linear regression. Univariate and multivariate analyses of the association of clinicopathological characteristics with patients’ OS and DFS were performed using the Cox proportional hazards model. The latter was adjusted for prognostic factors, including age, menopausal state, number of births, T stage, N stage, hormone receptors status, HER2 status, Ki67, p53 status, molecular subtype, endocrine therapy and radiation therapy. Survival curves were drawn using the Kaplan-Meier method. All statistical tests were two-tailed, and P values<0.05 were considered statistically significant.

## Results

### Patients’ baseline characteristics

The 221 patients included in this study were all women with a median age of 49 years. A total of 49 (22.2%) BC patients were included in the MetS group, and 172 (77.8%) BC patients were in the non-MetS group. Compared to the non-MetS group, MetS group patients were more likely to be older (P<0.001) and postmenopausal (P<0.001), and the MetS group included a higher proportion of Ki-67≤14 (P=0.024) patients and more childbirths (P=0.014). Body weight, BMI, FBG, TG, TC, LDL-C, and blood pressure were higher and HDL-C levels were lower in the MetS group than in the non-MetS group, and all these differences were statistically significant. MetS status was not associated with clinical T stage, N stage, hormone receptors, HER2 status or p53 status, and no differences in the number of NAC dose reductions and treatment interruptions were noted between the two groups (all P>0.05) (**Table 1**).

**Table 1:** Patient clinicopathological characteristics by MetS status.

Variable	Total (n=221)	MetS group (n=49)	Non-MetS group (n=172)	P
Age (years)	49.190 ± 9.415	54.730 ± 8.129	47.610 ± 9.174	<0.001
Menopause				<0.001
No	128 (57.9%)	16 (32.7%)	112 (65.1%)	
Yes	93 (42.1%)	33 (67.3%)	60 (34.9%)	
Number of births				0.014
0	27 (12.2%)	2 (4.1%)	25 (14.5%)	
1	137 (62.0%)	28 (57.1%)	109 (63.4%)	
2	44 (19.9%)	17 (34.7%)	27 (15.7%)	
>2	13 (5.9%)	2 (4.1%)	11 (6.4%)	
T Stage				0.138
cT_1_	31 (14.0%)	10 (20.4%)	21 (12.2%)	
cT_2_	143 (64.7%)	28 (57.2%)	115 (66.9%)	
cT_3_	41 (18.6%)	8 (16.3%)	33 (19.2%)	
cT_4_	6 (2.7%)	3 (6.1%)	3 (1.7%)	
N Stage				0.134
N_0_	11 (5.0%)	3 (6.1%)	8 (4.6%)	
N_1_	36 (16.3%)	4 (8.2%)	32 (18.6%)	
N_2_	75 (33.9%)	14 (28.6%)	61 (35.5%)	
N_3_	99 (44.8%)	28 (57.1%)	71 (41.3%)	
Hormone receptors				0.612
ER+/PR+	90 (40.7%)	22 (44.9%)	68 (39.5%)	
ER+/PR-	31 (14.0%)	8(16.3%)	23 (13.4%)	
ER-/PR-	94 (42.5%)	18 (36.7%)	76 (44.2%)	
HER2				0.127
Negative	142 (64.3%)	36 (73.5%)	106 (61.6%)	
Positive	79 (35.7%)	13 (26.5%)	66 (38.4%)	
Ki-67(%)				0.024
≤14	58 (26.2%)	19 (38.8%)	39 (22.7%)	
>14	163 (73.8%)	30 (61.2%)	133 (77.3%)	
p53				0.954
Negative	130 (58.8%)	29 (59.2%)	101 (58.7%)	
Positive	91 (41.2%)	20 (40.8%)	71 (41.3%)	
Subtype				0.550
Luminal A	21 (9.5%)	7 (14.3%)	14 (8.1%)	
Luminal B	106 (48.0%)	24 (49.0%)	82 (47.7%)	
HER2-E	50 (22.6%)	9 (18.4%)	41 (23.8%)	
TNBC	44 (19.9%)	9 (18.4%)	35 (20.4%)	
NAC dose reduction				0.626
No	194 (87.8%)	44 (89.8%)	150 (87.2%)	
Yes	27 (12.2%)	5 (10.2%)	22 (12.8%)	
NAC treatment interruption				0.294
No	144 (65.2%)	30 (61.2%)	119 (69.2%)	
Yes	77 (34.8%)	19 (38.8%)	53 (30.8%)	
Height (cm)	160.102 ± 5.322	159.306 ± 6.249	160.328 ± 5.024	0.296
Weight (kg)	62.887 ± 9.260	67.602 ± 8.598	61.544 ± 9.021	<0.001
BMI (kg/m2)	24.524 ± 3.309	26.617 ± 2.818	23.927 ± 3.200	<0.001
FBG (mmol/L)	5.300 (4.800-5.800)	6.100 (5.400-6.950)	5.100 (4.700-5.600)	<0.001
TG (mmol/L)	1.140 (0.785-1.550)	1.850 (1.340-2.780)	0.955 (0.730-1.320)	<0.001
TC (mmol/L)	4.693 ± 0.961	5.027 ± 1.017	4.597 ± 0.925	0.005
HDL-C (mmol/L)	1.620 ± 0.401	1.359 ± 0.341	1.695 ± 0.0.386	<0.001
LDL-C (mmol/L)	3.135 ± 0.890	3.440 ± 0.917	3.048 ± 0.865	0.006
SBP (mmHg)	124.411 ± 20.237	140.787 ± 18.057	119.746 ± 18.347	<0.001
DBP (mmHg)	75.813 ± 11.901	83.435 ± 12.856	73.641 ± 10.697	<0.001

MetS, metabolic syndrome; ER, estrogen receptor; PR, progesterone receptor; HER2, human epidermal growth factor receptor 2; HER2-E, HER2-enriched; TNBC, triple negative breast cancer; NAC, neoadjuvant chemotherapy; BMI, body mass index; FBG, fasting blood glucose; TG, triglycerides; TC, total cholesterol; HDL-C, high-density lipoprotein cholesterol; LDL-C, low-density lipoprotein cholesterol; SBP, systolic blood pressure; DBP, diastolic blood pressure.

### Univariate and multivariate analysis of pCR

**Figure 1:**
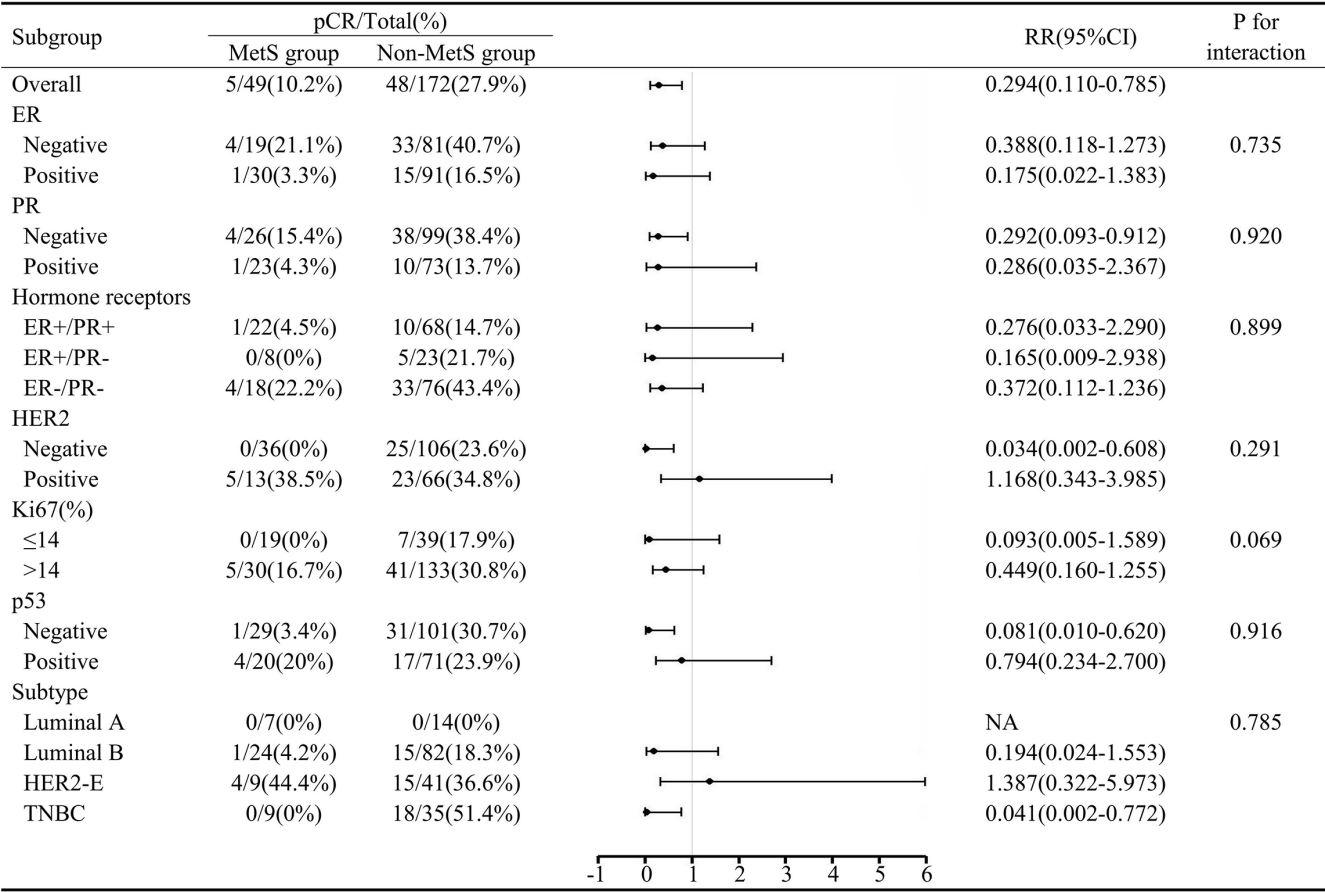
Subgroup analysis of MetS and pCR. MetS, metabolic syndrome; pCR, pathologic complete response; RR, risk ratio; CI, confidence interval; ER, estrogen receptor; PR, progesterone receptor; HER2, human epidermal growth factor receptor 2; HER2-E, HER2-enriched; TNBC, triple negative breast cancer.

In this study, a total of 53 (24.0%) patients achieved pCR after NAC, including five patients in the MetS group and 48 in the non-MetS group. Univariate analysis showed that the non-MetS group was more likely to achieve pCR than the MetS group (P=0.015). Patients who were hormone receptors negative, HER2 positive or Ki67>14% were more likely to achieve pCR (**Table 2**). Multivariate analysis showed that compared with ER+/PR+ patients, ER+/PR- patients and ER-/PR- patients had a higher probability of pCR, and ER-/PR- patients were particularly associated with pCR (OR=3.941, 95% CI: 1.772~8.766, P=0.001), and this finding reached statistical significance. Compared with the non-MetS group, it was more difficult for the MetS group to obtain pCR (OR=0.316, 95% CI: 0.113~0.886, P=0.028), indicating MetS and hormone receptors status were independent predictors of pCR (**Table 3**). Subgroup analysis showed that the relationship between MetS and pCR was more significant in the PR (−), HER2 (−), p53(−), and triple negative breast cancer (TNBC) subgroups (**Figure 1**).

**Table 2:** Univariate analysis between clinical characteristics and pCR.

Variable	Total (n=221)	pCR (n=53)	OR	CI (95%)	P
Age(years)					
≤49	115 (52.0%)	30 (56.6%)	Ref.	Ref.	Ref.
>49	106 (48.0%)	23 (43.4%)	0.785	0.422-1.462	0.446
Menopause					
No	128 (57.9%)	35 (66.0%)	Ref.	Ref.	Ref.
Yes	93 (42.1%)	18 (34.0%)	0.638	0.335-1.215	0.171
Number of births					
0	27 (12.2%)	7 (13.2%)	Ref.	Ref.	Ref.
1	137 (62.0%)	34 (64.1%)	0.943	0.367-2.424	0.903
2	44 (19.9%)	9 (17.0%)	0.735	0.237-2.275	0.593
>2	13 (5.9%)	3 (5.7%)	0.857	0.182-4.042	0.846
T Stage					
cT_1_+cT_2_	174 (78.7%)	46 (86.8%)	Ref.	Ref.	Ref.
cT_3_+cT_4_	47 (21.3%)	7 (13.2%)	0.487	0.204-1.163	0.105
N Stage					
N_0_	11 (5.0%)	2 (3.8%)	Ref.	Ref.	Ref.
N_1_+N_2_+N_3_	210 (95.0%)	51 (96.2%)	1.443	0.302-6.898	0.646
Hormone receptors					
ER+/PR+	90 (40.7%)	11 (20.8%)	Ref.	Ref.	Ref.
ER+/PR-	31 (14.0%)	5 (9.4%)	1.381	0.439-4.346	0.581
ER-/PR-	94 (42.5%)	37 (69.8%)	4.662	2.193-9.912	<0.001
HER2					
Negative	142 (64.3%)	25 (47.2%)	Ref.	Ref.	Ref.
Positive	79 (35.7%)	28 (52.8%)	2.569	1.366-4.832	0.003
Ki67(%)					
≤14	58 (26.2%)	7 (13.2%)	Ref.	Ref.	Ref.
>14	163 (73.8%)	46 (86.8%)	2.864	1.212-6.773	0.017
p53					
Negative	130 (58.8%)	32 (60.4%)	Ref.	Ref.	Ref.
Positive	91 (41.2%)	21 (39.6%)	0.919	0.489-1.725	0.792
MetS status					
No	172 (77.8%)	48 (90.6%)	Ref.	Ref.	Ref.
Yes	49 (22.2%)	5 (9.4%)	0.294	0.110-0.785	0.015

pCR, pathologic complete response; OR, odds ratio; CI, conﬁdence interval; ER, estrogen receptor; PR, progesterone receptor; HER2, human epidermal growth factor receptor 2; MetS, metabolic syndrome.

**Table 3:** Multivariate analysis between clinical characteristics and pCR.

Variable	OR	CI (95%)	P
Hormone receptors			
ER+/PR+	Ref.	Ref.	Ref.
ER+/PR-	1.334	0.404-4.403	0.636
ER-/PR-	3.941	1.772-8.766	0.001
HER2			
Negative	Ref.	Ref.	Ref.
Positive	1.545	0.770-3.099	0.221
Ki67(%)			
≤14	Ref.	Ref.	Ref.
>14	2.395	0.962-5.962	0.061
MetS status			
No	Ref.	Ref.	Ref.
Yes	0.316	0.113-0.886	0.028

pCR, pathologic complete response; OR, odds ratio; CI, conﬁdence interval; ER, estrogen receptor; PR, progesterone receptor; HER2, human epidermal growth factor receptor 2; MetS, metabolic syndrome.

### Changes in MetS after NAC

**Figure 2:**
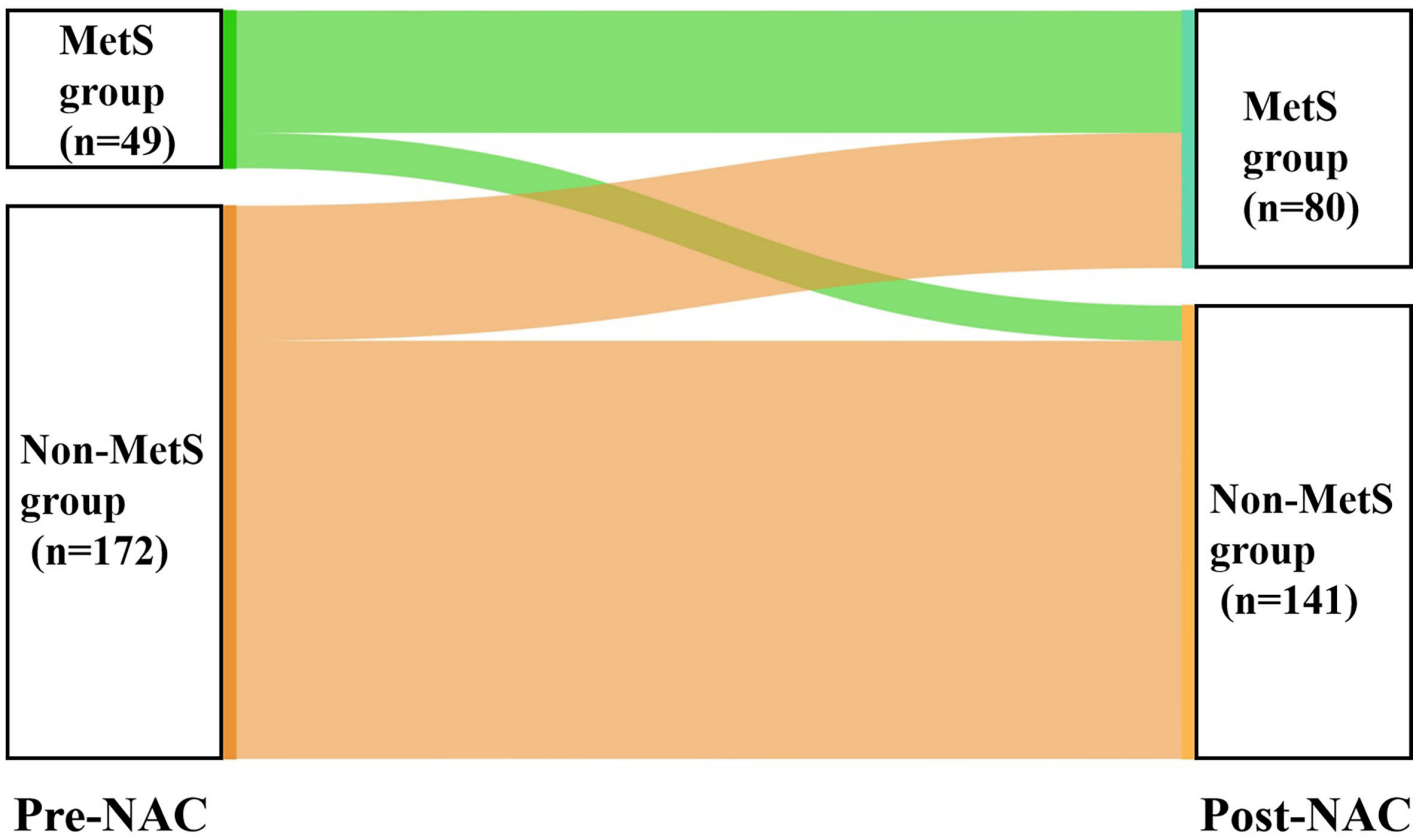
Changes in MetS status before and after NAC. MetS, metabolic syndrome; NAC, neoadjuvant chemotherapy.

The average duration of NAC was 4.67 months. After NAC, all metabolic parameters deteriorated, and the number of MetS components increased significantly. Among them, blood lipid indices, including TG, TC, HDL-C, and LDL-C, showed statistical deterioration (P<0.010) (**Table 4**). There were 49 (22.2%) patients in the MetS group before NAC and 80 (36.2%) patients in the MetS group after NAC. Forty-two (24.4%) patients in the non-MetS group met the diagnostic criteria for MetS after NAC (**Figure 2**).

**Table 4:** Changes in metabolic parameters before and after NAC.

Variable	Pre-NAC	Post-NAC	t/Z	P
Height (cm)	160.102 ± 5.322	–	–	–
Weight (kg)	62.887 ± 9.260	63.991 ± 9.389	-1.245	0.214
BMI (kg/m2)	24.524 ± 3.309	24.956 ± 3.370	-1.362	0.174
FBG (mmol/L)	5.300 (4.800-5.800)	5.400 (4.950-5.900)	-1.880	0.060
TG (mmol/L)	1.140 (0.785-1.550)	1.730 (1.245-2.490)	-8.054	<0.001
TC (mmol/L)	4.693 ± 0.961	5.172 ± 1.053	-5.002	<0.001
HDL-C (mmol/L)	1.620 ± 0.401	1.503 ± 0.392	3.101	0.002
LDL-C (mmol/L)	3.135 ± 0.890	3.572 ± 0.906	-5.121	<0.001
SBP (mmHg)	124.411 ± 20.237	126.883 ± 17.612	-1.369	0.172
DBP (mmHg)	75.813 ± 11.901	76.706 ± 12.441	-0.771	0.441
No. of MetS components	1.440 ± 1.308	2.030 ± 1.321	-4.704	<0.001

NAC, neoadjuvant chemotherapy; BMI, body mass index; FBG, fasting blood glucose; TG, triglycerides; TC, total cholesterol; HDL-C, high-density lipoprotein cholesterol; LDL-C; low-density lipoprotein cholesterol; SBP, systolic blood pressure; DBP, diastolic blood pressure; MetS, metabolic syndrome.

### Survival analysis

The mean OS and DFS values of 221 patients to the follow-up deadline were 96.75 and 87.46 months, respectively. The five-year survival rate of the MetS group was 64.6%, whereas that of the non-MetS group was 85.3%. In univariate analysis, MetS was associated with a greater than twofold increased risk of breast cancer mortality and recurrence (OR=2.463, 95% CI 1.391-4.363, P=0.002) (OR=2.213, 95% CI 1.336-3.668, P=0.002). Compared with postmenopausal patients, premenopausal patients had a longer OS and DFS (OR=2.316, 95% CI 1.315-4.079, P=0.004) (OR=1.792, 95% CI 1.108-2.898, P=0.017) (**Table 5**).

**Table 5:** Univariate analysis of hazards ratios for OS and DFS by clinical characteristics and MetS status.

Variable	OS			DFS		
	HR	CI (95%)	P	HR	CI (95%)	P
Age(years)						
≤49	Ref.	Ref.	Ref.	Ref.	Ref.	Ref.
>49	1.509	0.863-2.638	0.149	1.355	0.838-2.190	0.216
Menopause						
No	Ref.	Ref.	Ref.	Ref.	Ref.	Ref.
Yes	2.316	1.315-4.079	0.004	1.792	1.108-2.898	0.017
Number of births						
0	Ref.	Ref.	Ref.	Ref.	Ref.	Ref.
1	1.213	0.470-3.126	0.690	1.255	0.564-2.794	0.578
2	1.629	0.574-4.627	0.359	1.507	0.614-3.697	0.370
>2	1.299	0.310-5.436	0.720	0.912	0.236-3.528	0.894
T Stage						
cT_1_+cT_2_	Ref.	Ref.	Ref.	Ref.	Ref.	Ref.
cT_3_+cT_4_	1.301	0.691-2.448	0.415	1.182	0.674-2.073	0.560
N Stage						
N_0_	Ref.	Ref.	Ref.	Ref.	Ref.	Ref.
N_1_+N_2_+N_3_	2.786	0.385-20.181	0.311	0.615	0.247-1.529	0.296
Hormone receptors						
ER+/PR+	Ref.	Ref.	Ref.	Ref.	Ref.	Ref.
ER+/PR-	2.107	0.978-4.540	0.057	1.735	0.906-3.324	0.097
ER-/PR-	1.430	0.755-2.707	0.272	0.971	0.564-1.672	0.915
HER2						
Negative	Ref.	Ref.	Ref.	Ref.	Ref.	Ref.
Positive	1.478	0.845-2.583	0.171	1.102	0.672-1.808	0.701
Ki67(%)						
≤14	Ref.	Ref.	Ref.	Ref.	Ref.	Ref.
>14	1.334	0.683-2.605	0.399	1.318	0.742-2.341	0.346
p53						
Negative	Ref.	Ref.	Ref.	Ref.	Ref.	Ref.
Positive	1.409	0.809-2.456	0.226	1.194	0.737-1.932	0.472
Subtype						
Luminal A	Ref.	Ref.	Ref.	Ref.	Ref.	Ref.
Luminal B	2.591	0.614-10.946	0.195	2.708	0.741-5.831	0.165
HER2-E	2.408	0.534-10.868	0.253	1.363	0.439-4.225	0.592
TNBC	3.118	0.698-13.933	0.137	1.868	0.615-5.676	0.270
Endocrine therapy						
No	Ref.	Ref.	Ref.	Ref.	Ref.	Ref.
Yes	0.818	0.462-1.448	0.491	1.345	0.833-2.172	0.225
Radiation therapy						
No	Ref.	Ref.	Ref.	Ref.	Ref.	Ref.
Yes	1.081	0.616-1.896	0.786	1.012	0.625-1.638	0.961
MetS status						
No	Ref.	Ref.	Ref.	Ref.	Ref.	Ref.
Yes	2.463	1.391-4.363	0.002	2.213	1.336-3.668	0.002

OS, overall survival; DFS, disease-free survival; HR, hazard ratio; CI, conﬁdence interval; ER, estrogen receptor; PR, progesterone receptor; HER2, human epidermal growth factor receptor 2; HER2-E, HER2-enriched; TNBC, triple negative breast cancer; MetS, metabolic syndrome.

In the multivariate analysis, hazard ratios were adjusted for age, menopausal state, number of births, T stage, N stage, hormone receptors status, HER2 status, Ki67, p53 status, molecular subtype, endocrine therapy and radiation therapy. High TG (≥1.7 mmol/L) and low HDL-C (<1.3 mmol/L) were individually associated with an increased risk of death (OR=2.452, CI 95% 1.271-4.731, P=0.007) (OR=2.069, 95% CI 1.073-3.988, P=0.030). High TG (≥1.7 mmol/L), low HDL-C (<1.3 mmol/L) and hypertension were individually associated with an increased risk of relapse (OR=1.855, 95% CI 1.046-3.291, P=0.035) (OR=1.883, 95% CI 1.066-3.327, P=0.029) (OR=1.802, 95% CI 1.042-3.116, P=0.035) in multivariable-adjusted models. However, MetS remained the most significant predictor of disease progression and death after adjustment. MetS patients had a 2.587-fold increased risk of death (OR=2.587, 95% CI 1.359-4.924, P=0.004) and a 2.228-fold increased risk of recurrence (OR=2.228, 95% CI 1.251-3.970, P=0.007) compared with patients who were not diagnosed with MetS. Compared to individuals without any component of MetS present, the risk of death and disease progression increased steeply as the number of MetS components increased. Patients with 1-2, 3, 4, and 5 components had a 1.763-, 2.865-, 6.304-, and 15.488-fold higher risk of death and a 1.951-, 2.995-, 4.584-, and 12.129-fold higher risk of relapse, respectively, than patients with 0 components (**Table 6**).

**Table 6:** Multivariate analysis of hazards ratios for OS and DFS by MetS components.

Variable	OS			DFS		
	HR	CI (95%)	P	HR	CI (95%)	P
MetS status						
No	Ref.	Ref.	Ref.	Ref.	Ref.	Ref.
Yes	2.587	1.359-4.924	0.004	2.228	1.251-3.970	0.007
BMI (kg/m2)						
<25	Ref.	Ref.	Ref.	Ref.	Ref.	Ref.
≥25	1.548	0.852-2.813	0.151	1.609	0.961-2.693	0.071
FBG (mmol/L)						
<6.1	Ref.	Ref.	Ref.	Ref.	Ref.	Ref.
≥6.1	1.902	0.981-3.687	0.057	1.687	0.905-3.147	0.100
TG (mmol/L)						
<1.7	Ref.	Ref.	Ref.	Ref.	Ref.	Ref.
≥1.7	2.452	1.271-4.731	0.007	1.855	1.046-3.291	0.035
HDL-C (mmol/L)						
≥1.3	Ref.	Ref.	Ref.	Ref.	Ref.	Ref.
<1.3	2.069	1.073-3.988	0.030	1.883	1.066-3.327	0.029
Hypertension						
No	Ref.	Ref.	Ref.	Ref.	Ref.	Ref.
Yes	1.639	0.883-3.041	0.117	1.802	1.042-3.116	0.035
Number of components						
0 components	Ref.	Ref.	Ref.	Ref.	Ref.	Ref.
1–2 components	1.763	0.750-4.144	0.194	1.951	0.949-4.009	0.069
3 components	2.865	1.036-7.922	0.042	2.995	1.250-7.176	0.014
4 components	6.304	1.692-23.492	0.006	4.584	1.335-15.734	0.016
5 components	15.488	3.282-73.083	0.001	12.129	2.833-51.921	0.001

OS, overall survival; DFS, disease-free survival; HR, hazard ratio; CI, conﬁdence interval; MetS, metabolic syndrome; BMI, body mass index; FBG, fasting blood glucose; TG, triglycerides; HDL-C, high-density lipoprotein cholesterol.

Multivariate analysis adjusted for age, menopausal state, number of births, T Stage, N Stage, hormone receptors status, HER2 status, Ki67, p53 status, molecular subtype, endocrine therapy and radiation therapy.

**Figure 3:**
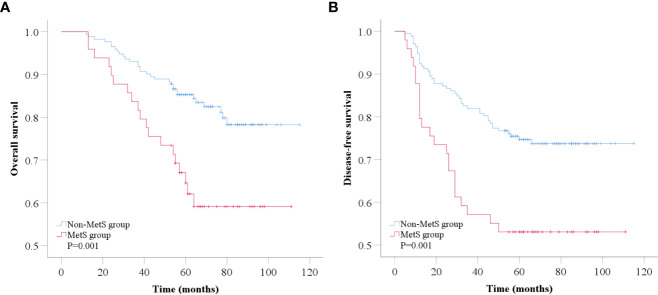
Kaplan-Meier analysis of overall survival **(A)** and disease-free survival **(B)** according to MetS status. MetS, metabolic syndrome.

**Figure 4:**
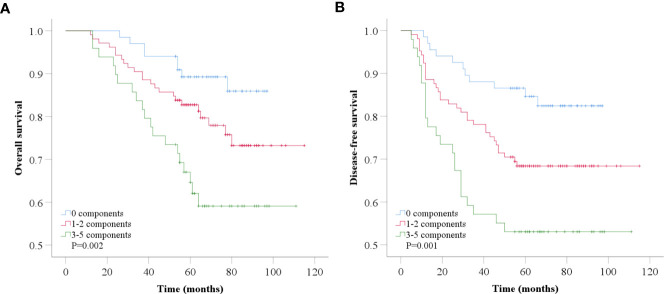
Kaplan-Meier analysis of overall survival **(A)** and disease-free survival **(B)** according to number of MetS components.

The follow-up time ranged from 12 to 115 months. The median follow-up time of 221 patients was 72.00 ± 2.44 months (6 years). Six years after diagnosis, the rates for OS and DFS were 84.4% vs. 59.1% (P=0.001) (**Figure 3A**) and 74.7% vs. 53.1% (P=0.001) (**Figure 3B**), respectively, in patients with non-MetS vs. MetS. Specifically, rates for OS and DFS were 85.9% vs. 77.9% vs. 59.1% (P=0.002) (**Figure 4A**) and 82.4% vs. 68.4% vs. 53.1% (P=0.001) (**Figure 4B**) in patients with 0 vs. 1-2 vs. 3-5 components of MetS. Kaplan−Meier survival analysis showed that BC patients receiving NAC with MetS before treatment had worse OS and DFS than those without MetS, and the difference was statistically significant.

## Discussion

As a significant public health problem worldwide, MetS is a cluster of risk factors for CVD and various malignant tumors. Several cohort studies and meta-analyses have highlighted the link between MetS and the prevalence, recurrence, and mortality of BC (11, 22, 23). NAC is increasingly being utilized as the first-line therapy for BC (6). Some studies have found that metabolic dysregulation status has predictive value for NAC in BC; specifically, higher BMI was associated with worse pCR to NAC (15). Diabetes and high FPG levels may be predictive of nonresponse to neoadjuvant chemotherapy in patients with BC (17). We evaluated the predictive effect of MetS on pCR in BC patients who received NAC, as it could be used to select those patients who demonstrate the most benefit from neoadjuvant systemic therapy, and analyzed long-term prognostic characteristics in these patients. To the best of our knowledge, our study is the first to date to systematically address the effect of MetS and its components on BC patients who received NAC.

Our paper retrospectively analyzed 221 BC patients who received NAC at Harbin Medical University Cancer Hospital. Similar to that noted other reports (14, 23), our study found that the MetS group included more elderly and postmenopausal patients than the non-MetS group. Unlike previous studies showing that MetS was associated with adverse pathological features (24), we found that the MetS group had a higher proportion of Ki-67≤14 patients. This finding may be due to the notion that lower Ki67 expression is associated with decreased metabolic activity, but more research is needed to reveal specific mechanisms (25). The MetS group had more childbirths than the non-MetS group. This is probably due to pregnancy involving marked alterations in metabolic parameters, including reduced insulin sensitivity in peripheral tissues, increased production of insulin from the pancreas, and accumulation and redistribution of body fat (26, 27). Previous studies showed that an increased number of births was associated with type 2 diabetes (28, 29).

In our study, 53 (24.0%) patients achieved pCR after NAC. The multivariate analysis showed that MetS (P=0.028) and hormone receptors status were independent predictors of pCR after NAC in breast cancer. Compared with the non-MetS group, the MetS group had more difficulty obtaining pCR. ER-/PR- patients had a higher probability of pCR than ER+/PR+ patients. In the subgroup analysis, we found that in the PR (−), HER2 (−), p53(−) and TNBC subgroups, MetS intervention can improve the pCR rate more effectively. This information should be considered when selecting patients who are most likely to benefit from NAC. Given that multicollinearity exists between hormone receptors and subtypes, the latter was not included in logistic regression models for analysis. Consistent with the extremely low pCR rates (0.3%) reported in previous studies (30), no luminal A patients obtained pCR in our study. The relationship between MetS and pCR in BC patients who underwent NAC was not consistent in previous studies. A study of 150 breast cancer neoadjuvant chemotherapy (BCNACT) patients which adopted International Diabetes Federation (IDF) criteria to diagnose MetS reported that MetS before BCNACT predicted a lower pCR rate (P=0.003) (31). Tong et al. found that in HER2-positive BC patients receiving neoadjuvant therapy, MetS showed a tendency to interfere with NAC effcacy, but the difference was not statistically significant in multivariate analysis (32). Similarly, Alan et al. did not identify a relationship between MetS and pCR in a study of 55 patients (33).

After NAC, all metabolic parameters worsened to varying degrees, and the number of MetS components was significantly increased (P<0.001). We can learn how quickly metabolic changes occur during NAC in BC patients who do not have any severe comorbidities at the time of diagnosis. Consistent with Tong’s study (32), the major metabolic disturbances observed were impaired lipid metabolism after NAC. We found that all blood lipid indices, including TG, TC, HDL-C, and LDL-C, were significantly worsened (P<0.010) to a greater extent than other metabolic biomarkers. Dyslipidemia, especially elevated LDL-C levels, is the most important independent risk factor for atherosclerotic CVD (34). The mechanism of dyslipidemia after NAC is unclear. Studies have shown that doxorubicin can regulate a series of genes involved in lipoprotein metabolism in liver cells, such as adenosine triphosphate (ATP)-binding cassette transporter A1 (ABCA1) and apoA1. In addition, doxorubicin and paclitaxel increase apoB protein levels, and paclitaxel decreases low-density lipoprotein receptor (LDLR) protein levels (35). This result suggests that long-term management of blood lipid profiles is necessary for BC patients who have received NAC, especially in patients who also require endocrine therapy, such as tamoxifen and aromatase inhibitors, which could alter lipid profiles in different ways (36, 37).

In survival analysis, we evaluated the association between MetS and its components with clinical outcomes in BC patients receiving NAC. By combining the results of multivariable adjusted data, our study showed that MetS was associated with higher overall mortality (P=0.004) and recurrence risk (P=0.007) in BC patients who received NAC, and this association was independent of some known prognostic factors, such as age, disease stage, and hormone receptors status. These results strongly indicated that MetS remains an independent predictor of poor prognosis in BC patients receiving NAC. In our study, even the presence of a single component of MetS was associated with an increased risk of recurrence and mortality in BC patients receiving NAC. In addition, as the number of MetS components increased, the risk of recurrence and mortality increased significantly. We observed that the risk of mortality increased from approximately twofold to greater than 15-fold among patients in whom the number of MetS components increased from 1 to 5 compared with those with no MetS components. Interestingly, among patients without MetS, the risk of recurrence mortality increased significantly as the number of MetS components increased. These results indicate that the greater the extent of metabolic dysregulation, the worse the outcomes in BC patients receiving NAC. This findings is consistent with Berrino’s study in early-stage breast cancer (14). Our study also investigated the impact of individual MetS components on BC outcome with differing results. However, as a comprehensive indicator, MetS was a more precise indicator of prognosis than individual MetS components.

The potential mechanisms between MetS and poor prognosis in breast cancer are currently under exploration. MetS itself is not a disease but a series of interdependent abnormal metabolic factors. Each of the metabolic alterations may be associated with the more aggressive tumor biology of BC. Insulin resistance (IR) and hyperinsulinemia are essential to the pathogenesis of type 2 diabetes and obesity (38). Insulin directly promotes breast tissue and tumor cell proliferation, thus possibly promoting BC incidence. In addition, hyperinsulinemia increases insulin-like growth factor 1 (IGF-1) bioavailability by increasing hepatic growth hormone receptor expression and repressing hepatic production of IGF-binding proteins (IGFBP) (39), resulting in hyperactivation of the Ras-MAPK and PI3K/Akt pathways in malignant cells to promote cell proliferation (40). Chronic inflammation, another critical pathophysiological feature of MetS (41), is also involved in the development and aggression of many malignancies. This process is characterized by reduced levels of anti-inflammatory cytokines (such as adiponectin) and high levels of pro-inflammatory cytokines (42). Adiponectin promotes glucose and fatty acid metabolism and improves insulin sensitivity and resistance. Adiponectin induces cell cycle arrest and apoptosis, increases the expression of the proapoptotic genes BAD (BCL2-associated agonist of cell death) and TP53 (tumor protein p53), decreases the antiapoptotic gene BCL2, and reduces the expression of CCND1 (cyclin D1) and CCNE2 (cyclin E2) in breast cancer cells, thereby inhibiting growth, invasion, and migration and inducing apoptosis of cancer cells (43). As the aromatase enzyme synthesizes estrogens in adipose tissue from circulating androgens, obesity could promote estrogen production (40), especially estradiol. This process also reduces adiponectin production, thereby attenuating the antitumor effect of adiponectin (44). Similarly, adiponectin levels are reduced in patients with diabetes and coronary heart disease. Furthermore, cholesterol promotes tumor growth and metastasis in BC through the PI3K/Akt signaling pathway (45). The mechanisms of the different molecular pathways involved in MetS and poor prognosis in patients with BC deserve further investigation.

Several limitations of this study should be noted. First, as a single-center study, our samples are obtained from single provinces in China, which may increase the heterogeneity between samples. The conclusion from this study needs to be verified in a larger and racially diverse population. Second, the diagnosis of MetS in our study was based on NCEP-ATPIII criteria. However, as a retrospective study, we did not have waist circumference data of patients, so we replaced waist circumference with BMI, which is more consistent with the actual Chinese characteristics. Third, we did not have medical treatment information for hyperglycemia, dyslipidemia, and hypertension in patients with MetS; thus, the number of patients with MetS was underestimated. In addition, this study did not exclude the interference from targeted therapy in the assessment of response to NAC in BC patients. In our study, only 17 (21.5%) of HER2-positive BC patients received targeted therapy with trastuzumab before surgery due to financial limitations. Trastuzumab has been covered by insurance in China only since 2017, and this information should be considered in further studies.

## Conclusion

In BC patients who received NAC, MetS was associated with poor outcomes, including a lower pCR rate and increased risk of recurrence and mortality, suggesting that timely MetS intervention is needed for a better prognosis.

## Data availability statement

The data analyzed in this study is subject to the following licenses/restrictions. The data that support the findings of this study are available from Harbin Medical University Cancer Hospital, but restrictions apply to the availability of these data, which were used under license for the current study, and so are not publicly available. 

## Ethics statement

The studies involving human participants were reviewed and approved by Ethics committee of Harbin Medical University Cancer Hospital. The patients/participants provided their written informed consent to participate in this study.

## Author contributions

ZZ, YZ, YH and SC contributed to the conception and design. ZZ, YL, CJ, YW and LS collected and analyzed the data and wrote the manuscript. All authors revised the manuscript critically. All authors contributed to the article and approved the submitted version.

## References

[1] Sung H Ferlay J Siegel RL Laversanne M Soerjomataram I Jemal A. Global cancer statistics 2020: Globocan estimates of incidence and mortality worldwide for 36 cancers in 185 countries. CA Cancer J Clin (2021) 71(3):209–49. doi: 10.3322/caac.21660 33538338

[2] Li T Mello-Thoms C Brennan PC. Descriptive epidemiology of breast cancer in China: Incidence, mortality, survival and prevalence. Breast Cancer Res Treat (2016) 159(3):395–406. doi: 10.1007/s10549-016-3947-0 27562585

[3] Hendrick RE Baker JA Helvie MA. Breast cancer deaths averted over 3 decades. Cancer (2019) 125(9):1482–8. doi: 10.1002/cncr.31954 30740647

[4] Shien T Iwata H. Adjuvant and neoadjuvant therapy for breast cancer. Jpn J Clin Oncol (2020) 50(3):225–9. doi: 10.1093/jjco/hyz213 32147701

[5] Sun C Shi L Gu Y Hu Y Wang J Liu Y. Clinical effects of neoadjuvant chemotherapy in treating breast cancer. Cancer Biother Radiopharm (2021) 36(2):174–9. doi: 10.1089/cbr.2019.3545 32343602

[6] Spring LM Fell G Arfe A Sharma C Greenup R Reynolds KL. Pathologic complete response after neoadjuvant chemotherapy and impact on breast cancer recurrence and survival: A comprehensive meta-analysis. Clin Cancer Res (2020) 26(12):2838–48. doi: 10.1158/1078-0432.CCR-19-3492 PMC7299787 32046998

[7] Wang H Mao X. Evaluation of the efficacy of neoadjuvant chemotherapy for breast cancer. Drug Des Devel Ther (2020) 14:2423–33. doi: 10.2147/DDDT.S253961 PMC7308147 32606609

[8] Lemieux I Després J-P. Metabolic syndrome: Past, present and future. Nutrients (2020) 12(11):3501. doi: 10.3390/nu12113501 PMC7696383 33202550

[9] Patnaik JL Byers T DiGuiseppi C Dabelea D Denberg TD. Cardiovascular disease competes with breast cancer as the leading cause of death for older females diagnosed with breast cancer: A retrospective cohort study. Breast Cancer Res (2011) 13(3):R64. doi: 10.1186/bcr2901 21689398 PMC3218953

[10] Abdel-Qadir H Austin PC Lee DS Amir E Tu JV Thavendiranathan P. A population-based study of cardiovascular mortality following early-stage breast cancer. JAMA Cardiol (2017) 2(1):88–93. doi: 10.1001/jamacardio.2016.3841 27732702

[11] Esposito K Chiodini P Colao A Lenzi A Giugliano D. Metabolic syndrome and risk of cancer: A systematic review and meta-analysis. Diabetes Care (2012) 35(11):2402–11. doi: 10.2337/dc12-0336 PMC3476894 23093685

[12] Agnoli C Berrino F Abagnato CA Muti P Panico S Crosignani P. Metabolic syndrome and postmenopausal breast cancer in the ordet cohort: A nested case-control study. Nutr Metab Cardiovasc Dis (2010) 20(1):41–8. doi: 10.1016/j.numecd.2009.02.006 PMC2819536 19361966

[13] Dong S Wang Z Shen K Chen X. Metabolic syndrome and breast cancer: Prevalence, treatment response, and prognosis. Front Oncol (2021) 11:629666. doi: 10.3389/fonc.2021.629666 33842335 PMC8027241

[14] Berrino F Villarini A Traina A Bonanni B Panico S Mano MP. Metabolic syndrome and breast cancer prognosis. Breast Cancer Res Treat (2014) 147(1):159–65. doi: 10.1007/s10549-014-3076-6 25104441

[15] Litton JK Gonzalez-Angulo AM Warneke CL Buzdar AU Kau S-W Bondy M. Relationship between obesity and pathologic response to neoadjuvant chemotherapy among women with operable breast cancer. J Clin Oncol (2008) 26(25):4072–7. doi: 10.1200/JCO.2007.14.4527 PMC6557586 18757321

[16] Hilvo M Gade S Hyötyläinen T Nekljudova V Seppänen-Laakso T Sysi-Aho M. Monounsaturated fatty acids in serum triacylglycerols are associated with response to neoadjuvant chemotherapy in breast cancer patients. Int J Cancer (2014) 134(7):1725–33. doi: 10.1002/ijc.28491 24114462

[17] Arici S Geredeli C Secmeler S Cekin R Sakin A Cihan S. The effects of diabetes and fasting plasma glucose on treatment of breast cancer with neoadjuvant chemotherapy. Curr Probl In Cancer (2020) 44(1):100485. doi: 10.1016/j.currproblcancer.2019.05.007 31200961

[18] Expert Panel on Detection, Evaluation, and Treatment of High Blood Cholesterol in Adults. Executive summary of the third report of the national cholesterol education program (Ncep) expert panel on detection, evaluation, and treatment of high blood cholesterol in adults (Adult treatment panel iii). JAMA (2001) 285(19):2486–97. doi: 10.1001/jama.285.19.2486 11368702

[19] Abbasi F Malhotra D Mathur A Reaven GM Molina CR. Body mass index and waist circumference associate to a comparable degree with insulin resistance and related metabolic abnormalities in south Asian women and men. Diabetes Vasc Dis Res (2012) 9(4):296–300. doi: 10.1177/1479164111433578 22278736

[20] Oda E Kawai R. Comparison among body mass index (Bmi), waist circumference (Wc), and percent body fat (%Bf) as anthropometric markers for the clustering of metabolic risk factors in Japanese. Intern Med (2010) 49(15):1477–82. doi: 10.2169/internalmedicine.49.3363 20686277

[21] Zhong Y Hu M Wang Q Yang Z Zhu N Wang F. The prevalence and related factors of metabolic syndrome in outpatients with first-episode drug-naive major depression comorbid with anxiety. Sci Rep (2021) 11(1):3324. doi: 10.1038/s41598-021-81653-2 33558554 PMC7870819

[22] Uzunlulu M Telci Caklili O Oguz A. Association between metabolic syndrome and cancer. Ann Nutr Metab (2016) 68(3):173–9. doi: 10.1159/000443743 26895247

[23] Buono G Crispo A Giuliano M De Angelis C Schettini F Forestieri V. Metabolic syndrome and early stage breast cancer outcome: Results from a prospective observational study. Breast Cancer Res Treat (2020) 182(2):401–9. doi: 10.1007/s10549-020-05701-7 PMC7297840 32500397

[24] Healy LA Ryan AM Carroll P Ennis D Crowley V Boyle T. Metabolic syndrome, central obesity and insulin resistance are associated with adverse pathological features in postmenopausal breast cancer. Clin Oncol (R Coll Radiol) (2010) 22(4):281–8. doi: 10.1016/j.clon.2010.02.001 20189371

[25] Jahani M Shahlaei M Norooznezhad F Miraghaee SS Hosseinzadeh L Moasefi N. Tsga10 over expression decreases metastasic and metabolic activity by inhibiting hif-1 in breast cancer cells. Arch Med Res (2020) 51(1):41–53. doi: 10.1016/j.arcmed.2019.12.002 32086108

[26] Stuebe AM Mantzoros C Kleinman K Gillman MW Rifas-Shiman S Seely EW. Gestational glucose tolerance and maternal metabolic profile at 3 years postpartum. Obstet Gynecol (2011) 118(5):1065–73. doi: 10.1097/AOG.0b013e3182325f5a PMC3268071 22015874

[27] Gunderson EP. Childbearing and obesity in women: Weight before, during, and after pregnancy. Obstet Gynecol Clin North Am (2009) 36(2):317–32. doi: 10.1016/j.ogc.2009.04.001 PMC2930888 19501316

[28] Araneta MRG Barrett-Connor E. Grand multiparity is associated with type 2 diabetes in Filipino American women, independent of visceral fat and adiponectin. Diabetes Care (2010) 33(2):385–9. doi: 10.2337/dc09-1477 PMC2809288 19918009

[29] Mueller NT Mueller NJ Odegaard AO Gross MD Koh WP Yuan JM. Higher parity is associated with an increased risk of type-ii diabetes in Chinese women: The Singapore Chinese health study. BJOG (2013) 120(12):1483–9. doi: 10.1111/1471-0528.12364 PMC4038299 23786390

[30] Haque W Verma V Hatch S Suzanne Klimberg V Brian Butler E Teh BS. Response rates and pathologic complete response by breast cancer molecular subtype following neoadjuvant chemotherapy. Breast Cancer Res Treat (2018) 170(3):559–67. doi: 10.1007/s10549-018-4801-3 29693228

[31] Lu Y Wang P Lan N Kong F Abdumijit A Tu S. Metabolic syndrome predicts response to neoadjuvant chemotherapy in breast cancer. Front Oncol (2022) 12:899335. doi: 10.3389/fonc.2022.899335 35847887 PMC9284232

[32] Tong Y-W Wang G Wu J-Y Huang O He J-R Zhu L. Insulin-like growth factor-1, metabolic abnormalities, and pathological complete remission rate in Her2-positive breast cancer patients receiving neoadjuvant therapy. Onco Targets Ther (2019) 12:3977–89. doi: 10.2147/OTT.S194981 PMC6535081 31190894

[33] Alan O Akin Telli T Aktas B Koca S Ökten IN Hasanov R. Is insulin resistance a predictor for complete response in breast cancer patients who underwent neoadjuvant treatment? World J Surg Oncol (2020) 18(1):242. doi: 10.1186/s12957-020-02019-y 32907593 PMC7488234

[34] Manthravadi S Shrestha A Madhusudhana S. Impact of statin use on cancer recurrence and mortality in breast cancer: A systematic review and meta-analysis. Int J Cancer (2016) 139(6):1281–8. doi: 10.1002/ijc.30185 27176735

[35] Sharma M Tuaine J McLaren B Waters DL Black K Jones LM. Chemotherapy agents alter plasma lipids in breast cancer patients and show differential effects on lipid metabolism genes in liver cells. PloS One (2016) 11(1):e0148049. doi: 10.1371/journal.pone.0148049 26807857 PMC4726544

[36] Wang X Zhu A Wang J Ma F Liu J Fan Y. Steroidal aromatase inhibitors have a more favorable effect on lipid profiles than nonsteroidal aromatase inhibitors in postmenopausal women with early breast cancer: A prospective cohort study. Ther Adv Med Oncol (2020) 12:1758835920925991. doi: 10.1177/1758835920925991 32518597 PMC7252381

[37] Alomar SA Găman M-A Prabahar K Arafah OA Almarshood F Baradwan S. The effect of tamoxifen on the lipid profile in women: A systematic review and meta-analysis of randomized controlled trials. Exp Gerontol (2022) 159:111680. doi: 10.1016/j.exger.2021.111680 34973347

[38] Chen Y Wen Y-y Li Z-r Luo D-l Zhang X-h. The molecular mechanisms between metabolic syndrome and breast cancer. Biochem Biophys Res Commun (2016) 471(4):391–5. doi: 10.1016/j.bbrc.2016.02.034 26891869

[39] Calle EE Kaaks R. Overweight, obesity and cancer: Epidemiological evidence and proposed mechanisms. Nat Rev Cancer (2004) 4(8):579–91. doi: 10.1038/nrc1408 15286738

[40] Khandekar MJ Cohen P Spiegelman BM. Molecular mechanisms of cancer development in obesity. Nat Rev Cancer (2011) 11(12):886–95. doi: 10.1038/nrc3174 22113164

[41] Mendonça FM de Sousa FR Barbosa AL Martins SC Araújo RL Soares R. Metabolic syndrome and risk of cancer: Which link? Metabolism (2015) 64(2):182–9. doi: 10.1016/j.metabol.2014.10.008 25456095

[42] Hauner D Hauner H. Metabolic syndrome and breast cancer: Is there a link? Breast Care (Basel) (2014) 9(4):277–81. doi: 10.1159/000365951 PMC4209278 25404888

[43] Chung SJ Nagaraju GP Nagalingam A Muniraj N Kuppusamy P Walker A. Adipoq/Adiponectin induces cytotoxic autophagy in breast cancer cells through Stk11/Lkb1-mediated activation of the ampk-Ulk1 axis. Autophagy (2017) 13(8):1386–403. doi: 10.1080/15548627.2017.1332565 PMC5584870 28696138

[44] Zhao P Xia N Zhang H Deng T. The metabolic syndrome is a risk factor for breast cancer: A systematic review and meta-analysis. Obes Facts (2020) 13(4):384–96. doi: 10.1159/000507554 PMC7590763 32698183

[45] Alikhani N Ferguson RD Novosyadlyy R Gallagher EJ Scheinman EJ Yakar S. Mammary tumor growth and pulmonary metastasis are enhanced in a hyperlipidemic mouse model. Oncogene (2013) 32(8):961–7. doi: 10.1038/onc.2012.113 PMC4063440 22469977

